# Effectiveness of Ultraviolet-C Light and a High-Level Disinfection Cabinet for Decontamination of N95 Respirators

**DOI:** 10.20411/pai.v5i1.372

**Published:** 2020-05-02

**Authors:** Jennifer L. Cadnum, Daniel F. Li, Sarah N. Redmond, Amrita R. John, Basya Pearlmutter, Curtis J. Donskey

**Affiliations:** 1 Research Service; Louis Stokes Cleveland VA Medical Center; Cleveland, Ohio; 2 Geriatric Research, Education, and Clinical Center; Louis Stokes Cleveland VA Medical Center; Cleveland, Ohio; 3 Department of Medicine; University Hospitals Cleveland Medical Center; Cleveland, Ohio; 4 Case Western Reserve University School of Medicine; Cleveland, Ohio

**Keywords:** Ultraviolet-C, decontamination, N95 respirator, peracetic acid, SARS-CoV-2

## Abstract

**Background:**

Shortages of personal protective equipment (PPE) including N95 respirators are an urgent concern in the setting of the global COVID-19 pandemic. Decontamination of PPE could be useful to maintain adequate supplies, but there is uncertainty regarding the efficacy of decontamination technologies.

**Methods:**

A modification of the American Society for Testing and Materials standard quantitative carrier disk test method (ASTM E-2197-11) was used to examine the effectiveness of 3 methods, including ultraviolet-C (UV-C) light, a high-level disinfection cabinet that generates aerosolized peracetic acid and hydrogen peroxide, and dry heat at 70°C for 30 minutes. We assessed the decontamination of 3 commercial N95 respirators inoculated with methicillin-resistant *Staphylococcus aureus* (MRSA) and bacteriophages MS2 and Phi6; the latter is an enveloped RNA virus used as a surrogate for coronaviruses. Three and 6 log_10_ reductions on respirators were considered effective for decontamination and disinfection, respectively.

**Results:**

UV-C administered as a 1-minute cycle in a UV-C box or a 30-minute cycle by a room decontamination device reduced contamination but did not meet criteria for decontamination of the viruses from all sites on the N95s. The high-level disinfection cabinet was effective for decontamination of the N95s and achieved disinfection with an extended 31-minute cycle. Dry heat at 70°C for 30 minutes was not effective for decontamination of the bacteriophages.

**Conclusions:**

UV-C could be useful to reduce contamination on N95 respirators. However, the UV-C technologies studied did not meet pre-established criteria for decontamination under the test conditions used. The high-level disinfection cabinet was more effective and met criteria for disinfection with an extended cycle.

## INTRODUCTION

Personal protective equipment (PPE) is essential for protection of personnel and patients in healthcare settings [1]. The pandemic of coronavirus disease 2019 (COVID-19) caused by severe acute respiratory syndrome coronavirus 2 (SARS-CoV-2) has resulted in shortages of critical supplies, including PPE [2]. These shortages have led many facilities to consider strategies to extend or reuse PPE, particularly N95 filtering facepiece respirators. The Centers for Disease Control and Prevention and the National Institute for Occupational Safety and Health (NIOSH) have provided guidance on the acceptability of extended use or limited reuse of N95 respirators [3]. The guidance document includes a discussion of potential concerns regarding these practices, particularly the risk for contact transmission from touching a contaminated respirator [3].

Decontamination of N95 respirators and other PPE has been widely discussed as a potential strategy to maintain adequate supplies in crisis situations [4–9]. A variety of decontamination methods have been proposed, including heat and technologies such as ultraviolet-C (UV-C) light and hydrogen peroxide vapor [4–9]. However, there is relatively limited published information on the efficacy of these technologies, and there is uncertainty regarding how to best deploy them (eg, decontamination after each patient interaction or once daily). There is also concern that decontamination technologies could alter the level of protection provided by PPE [10–11].

There is an urgent need for evidence regarding the effectiveness of decontamination strategies for PPE. The goal of the current study was to examine the effectiveness of UV-C light and a high-level disinfection cabinet for decontamination of N95 respirators. For UV-C light, we studied a 1-minute cycle delivered by a UV-C decontamination box that could potentially be used for rapid decontamination after each removal of a respirator and a 30-minute treatment delivered by a room decontamination device that might be used once daily. The high-level disinfection cabinet generates submicron droplets of aerosolized peracetic acid and hydrogen peroxide and has been shown to be very effective in eliminating microorganisms from hard surfaces in patient rooms and on portable equipment [12–13]. We hypothesized that the disinfection cabinet would be very effective while UV-C light might have reduced efficacy in contrast to previous reports that evaluated microorganism reductions on steel disk carriers or hard surfaces in healthcare settings [14–15]. Soft, irregular surfaces may present a challenge for UV-C decontamination due to the potential for areas of shadowing and absorption of organisms into sites with reduced delivery of UV-C [16].

## METHODS

Table 1 shows the test organisms studied and their characteristics. The enveloped double-stranded RNA virus bacteriophage Phi6 has been used as a surrogate for coronaviruses and influenza in previous studies [17]. The bacteriophages MS2 and Phi6 were propagated as previously described in *Escherichia coli* and *Pseudomonas syringae*, respectively [1, 17]. The other test organisms were prepared as previously described [12–13].

**Table 1.** Characteristics of the test organisms

Organism	Source	Characteristics
*Acinetobacter baumanii*	Clinical isolate	Non-spore-forming Gram-negative bacterium
Vancomycin-resistant *Enterococcus faecium*	Clinical isolate	VanB type Gram-positive non-spore-forming bacterium
NDM1-producing *Klebsiella pneumoniae*	ATCC BAA-2146	NDM1-producing non-spore-forming Gram-negative bacterium
Methicillin-resistant *Staphylococcus aureus* (MRSA)	Clinical isolate; pulsed-field gel electrophoresis type USA 400	Non-spore-forming Gram-positive bacterium
*Escherichia coli*	Clinical isolate	NDM1-producing Non-spore-forming Gram-negative bacterium
Bacteriophage Phi X174	ATCC 13706-B1	Nonenveloped single-stranded DNA virus (27 µm particle size)
Bacteriophage Phi6	HER 102	Enveloped, double-stranded RNA virus (85 µm particle size)
Bacteriophage MS2	ATCC 15597-B1	Nonenveloped, single-stranded RNA virus (26 µm particle size)
*Candida auris*	Centers for Disease Control and Prevention strain 0385	Emerging fungal pathogen that is often resistant to antifungal agents
*Candida albicans*	ATCC 10231	Yeast that commonly colonizes the gastrointestinal tract
*Clostridioides difficile*	ATCC 43598	Spore preparation
*Bacillus subtilis*	ATCC 6051	Spore-forming Gram-positive bacterium

Abbreviations: ATCC, American Type Culture Collection; HER, Félix d'Hérelle Reference Center for bacterial viruses of the Université Laval; NDM, New Delhi metallo-betalactamase.

### Efficacy of UV-C light against the test organisms on steel disk carriers

Initial testing was performed to assess the efficacy of UV-C against all the test organisms using a modification of the American Society for Testing and Materials standard quantitative carrier disk test method (ASTM E-2197-11) [18]. The purpose of this assessment was to determine the relative susceptibility of bacteriophages Phi6 and MS2 to UV-C in comparison to bacterial and fungal organisms listed in Table 1. For each pathogen, 10-μL aliquots containing ~10^6^ colony-forming units (CFU) or plaque-forming units (PFU) in phosphate-buffered saline were spread to cover steel disks 20 mm in diameter. The steel disk carriers were positioned 32 inches from a room decontamination device (Moonbeam 3, Diversey, Fort Mill, SC) that was operated for a 90-second cycle or 180-second cycle for more UV-C resistant organisms. The relatively short cycle times were chosen in order to allow a comparison in log_10_ reductions (ie, complete elimination of the inoculum was common for susceptible organisms at higher UV-C doses). The disks were positioned at a height of 3 feet and oriented vertically in parallel with the UV-C bulbs. The disks were processed as previously described [12–13]. Log_10_ CFU or PFU reductions were calculated by comparing recovery from UV-C-exposed carriers to untreated controls.

### Efficacy of UV-C light for decontamination of N95 respirators

**Figure 1.**
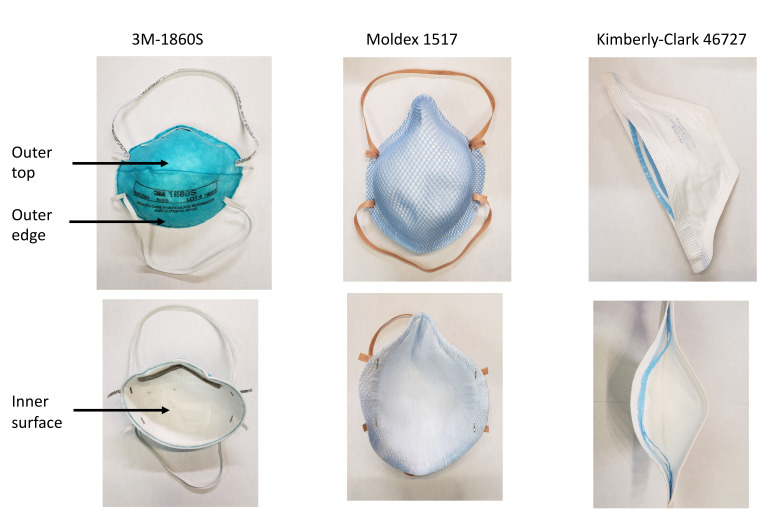
Pictures of the 3 N95 respirators evaluated in the study and areas where the test organisms were applied.

For testing of efficacy of UV-C against organisms on N95 respirators, we used the methicillin-resistant *Staphylococcus aureus* (MRSA) test strain and bacteriophages MS2 and Phi6. Three N95 respirators were studied, the 3M 1860S, Moldex 1517, and Kimberly-Clark 46727. Aliquots of 10 μL containing 10^6^ CFU or PFU of the test organisms were suspended in 8% simulated mucus prepared as previously described [19]. The test organisms were pipetted onto 3 different areas on the respirator surface (outer top, outer edge, and inner surface) (Figure 1). The suspensions were spread with a sterile loop to cover an area of 1 cm^2^ and allowed to air dry. The respirators were placed inside a UV-C box (Advanced Ultraviolet Systems, South Hill, VA) that provides decontamination with 1 lamp below and 1 above the item to be decontaminated. The UV-C cycle time for the UV-C box was 60 seconds.

Additional experiments were conducted with the inoculated Moldex 1517 N95 respirator positioned 3 feet from a low-pressure mercury UV room decontamination device (Optimum UV, Clorox Company, Oakland, CA). The device was operated for two cycles; 1 cycle of 15 minutes was performed with the exterior side of the mask facing the lamps and another 15-minute cycle was performed with the interior side of the respirator facing the lamps. For each UV-C experiment, a colorimetric indicator (UVC Dose Indicator, American Ultraviolet, Lebanon, IN) was placed adjacent to the respirator to provide a visual assessment of UV-C delivery to the respirator. 

After the UV-C treatments, the inoculated sections of the N95 respirators were cut out, vortexed for 1 minute in 1 mL of phosphate-buffered saline with 0.02% Tween, and serial dilutions were plated on selective media to quantify viable organisms. All tests were performed in triplicate. Log_10_ CFU or PFU reductions were calculated by comparing recovery from UV-C-exposed respirators to untreated control respirators.

### Efficacy of a multi-purpose high-level disinfection cabinet for decontamination of N95 respirators

The Multi-Purpose High-Level Disinfection Cabinet (Altapure, Mequon, WI) has been described previously [13]. On steel disk carriers, the cabinet was effective in eliminating ≥5 log_10_ PFU or CFU of *C. auris, Clostridioides difficile* spores, MRSA, and bacteriophage MS2 with a 21-minute total cycle time. This included 1 minute of fogging, 5 minutes of dwell time, and 15 minutes of scrubbing and dehumidification during which peracetic acid, hydrogen peroxide, and acetic acid were removed by passing through activated charcoal filters [13]. The cabinet also was effective in killing *Geobacillus stearothermophilus* biological indicator spores [13].

For testing of efficacy against organisms on an N95 respirator, we used the MRSA test strain and bacteriophage MS2 applied to Moldex 1517 respirators that were inoculated as described previously. We tested 1, 2, and 3 cycles of treatment with the cabinet to determine if longer treatment time would increase efficacy. Because multiple cycles increased efficacy, we also tested a single extended cycle with a 15-minute dwell time and 31-minute total cycle time. After completion of the treatment, log_10_ CFU or PFU reductions were calculated by comparing recovery from exposed respirators to untreated control respirators. The tests were performed in triplicate. For the testing with 3 cycles, additional testing was completed with the 2 other respirator types, and the suspension containing 10^6^ PFU of MS2 and 10^6^ CFU of MRSA was sprayed onto the entire inner and outer surface of the Moldex 1517 respirator. Finally, we tested the efficacy of a single 21-minute cycle against *C. difficile* spores (ATCC strain 43598), MRSA, and bacteriophage MS2.

### Efficacy of dry heat for decontamination of N95 respirators

Heat has been discussed as an alternative to other decontamination methods for SARS-CoV-2. Therefore, we examined the effectiveness of heating N95 respirators to 70°C for 30 minutes in an oven (Economy Incubator, Precision). N95 respirators were contaminated with MRSA and the bacteriophages by pipetting onto 3 different areas of the respirator as previously described, and log_10_ reductions were calculated.

## DATA ANALYSIS

There is no standard level of germicidal activity recommended for N95 respirators. Rutala *et al*. [20] have proposed that disinfectants that demonstrate a 3-log_10_ reduction on carriers are likely to be clinically effective on surfaces. For purposes of analysis, we considered a ≥3-log_10_ reduction in recovery of organisms inoculated onto N95 respirators to be effective for decontamination of respirators and a 6-log_10_ reduction to be effective for disinfection. Reductions greater than 6 log_10_ were rounded to 6-log_10_ reductions.

Analysis of variance (ANOVA) was performed to compare the mean log_10_ reductions for the different N95 respirators and for the organisms assessed in dry heat. ANOVA models adjusted for the additional experimental settings (side of mask, organism) while assessing the parameter of interest. All analyses were performed using R version 3.5.1 statistical software (The R Foundation for Statistical Computing, Vienna, Austria). 

## RESULTS

### Efficacy of UV-C light against the test organisms on steel disk carriers

Table 2 shows the comparison of log_10_ CFU or PFU reductions achieved on steel disk carriers by 90 or 180 seconds of exposure to UV-C from a room decontamination device. Bacteriophage MS2 and bacteriophage Phi6 were less susceptible to UV-C than vegetative bacteria such as MRSA, but more susceptible than *Candida* species and *C. difficile* spores.

**Table 2.** Comparison of log10 colony-forming unit (CFU) or plaque-forming unit (PFU) reductions achieved on steel disk carriers by 90 seconds or 180 seconds of exposure to UV-C from a room decontamination device.

Time (seconds)	Organism	Susceptible to UV-C	Log_10_ CFU reduction
90	*Acinetobacter baumanii*	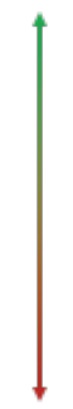	4.32
90	Vancomycin-resistant *Enterococcus faecium*		3.75
90	*Klebsiella pneumoniae*		3.69
90	MRSA		3.28
90	NDM1 *Escherichia coli*		3.27
90	PHI X174 (SS-DNA non-enveloped)		2.49
90	PHI 6 (DS-RNA Enveloped)		0.91
90	MS2 (SS-RNA non-enveloped)		0.84
180	*Candida auris*		1.13
180	*Candida albicans*		1.13
180	*Clostridioides difficile* spores		1.05
180	*Bacillus subtillus spores*		0.72
		Resistant to UV-C	

### Efficacy of UV-C light for decontamination of N95 respirators

**Figure 2.**
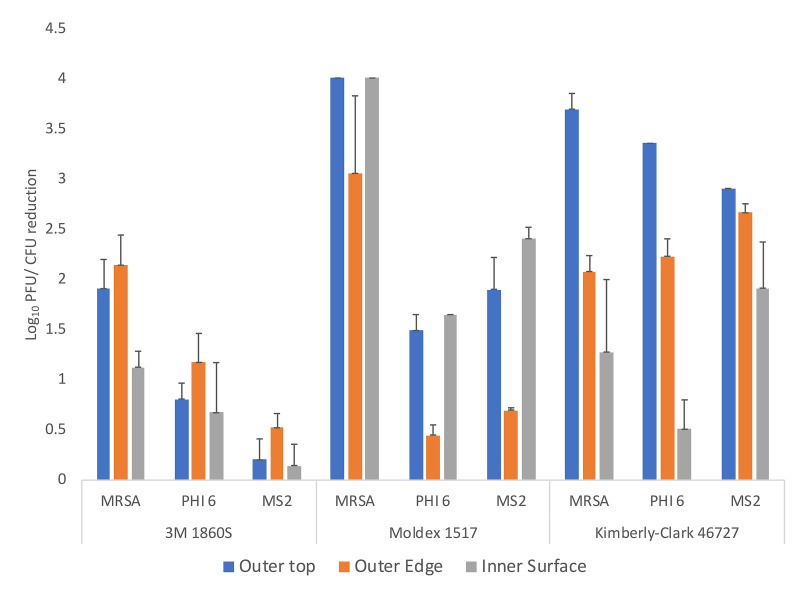
Efficacy of ultraviolet-C (UV-C) light for decontamination of methicillin-resistant *Staphylococcus aureus* (MRSA), bacteriophage MS2, and bacteriophage Phi6 on 3 different N95 respirators (3M 1860S, Moldex 1517, and Kimberly-Clark 46727). Aliquots of 10 μL containing 10^6^ colony-forming units (CFU) or plaque-forming units (PFU) of the test organisms in the simulated mucus suspension were spread to cover an area of 1 cm^2^ on 3 different areas on the respirator surface (top exterior, edge exterior, and interior) as shown in Figure 1. The respirators were exposed to a 60-second cycle of UV-C inside a UV-C box. Error bars indicate standard error.

Figure 2 shows the log reductions achieved on the 3 N95 respirators with the 1-minute cycle in the UV-C box for MRSA, bacteriophage MS2, and bacteriophage Phi6. Reductions in MRSA were consistently greater than reductions of the bacteriophages for most test sites. Reductions in MS2 and Phi6 were similar at most test sites. Differences in reduction across masks were statistically significant (F = 5.69, df = 2, *P* < 0.01). Reductions were consistently lower on the 3M 1860S N95 respirator in comparison with the other respirators. Reductions were also consistently lower for the interior surfaces of the respirators versus the exterior surfaces; it was noted that the exterior surfaces were impermeable to the liquid suspensions while the interior surfaces were not. For the 3M 1860S N95 respirator, the reductions achieved on the exterior edge were lower than the exterior top surface; for this respirator, the edge has a fold that might result in shadowing that could reduce UV-C delivery (Figure 1).

**Figure 3.**
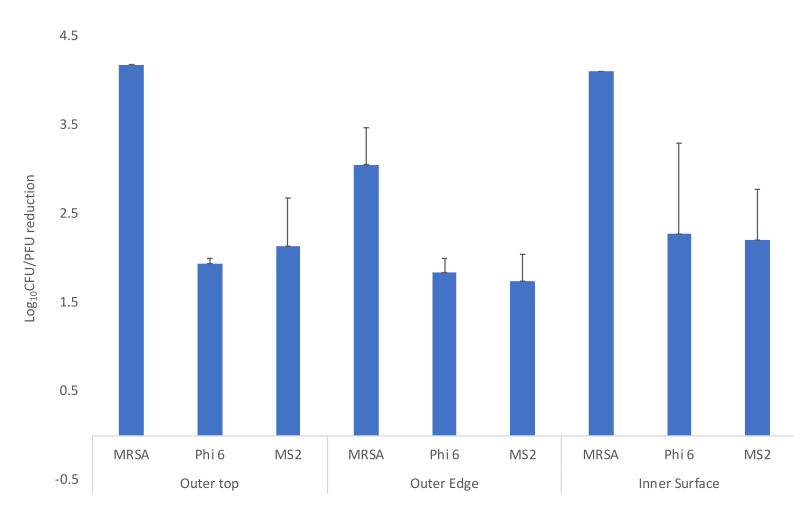
Efficacy of ultraviolet (UV) light delivered by low-pressure mercury decontamination device for decontamination of methicillin-resistant *Staphylococcus aureus* (MRSA), bacteriophage MS2, and bacteriophage Phi6 on Moldex 1517 N95 respirators. 10-μL aliquots containing 10^>6^ colony-forming units (CFU) or plaque-forming units (PFU) of the test organisms in the simulated mucous suspension were spread to cover an area of 1-cm2 on 3 different areas on the respirator surface (top exterior, edge exterior, and interior) as shown in Figure 1. The respirator was exposed to a 15-minute cycle of UV at 3 feet from the bulbs then turned for another 15-minute cycle on the opposite side. Error bars indicate standard error.

Figure 3 shows the efficacy of UV delivered by the room decontamination device with 2 15-minute cycles for the Moldex 1517 N95 respirator. In general, greater reductions were achieved with the longer UV-C cycles using the low-pressure mercury room decontamination device in comparison to the short cycles in the UV-C box, and reductions on the interior surfaces and the exterior surface were comparable. No visible changes were observed in any of the respirators after 3 or more treatment cycles with the UV-C room decontamination device or with the UV-C box.

**Figure 4.**
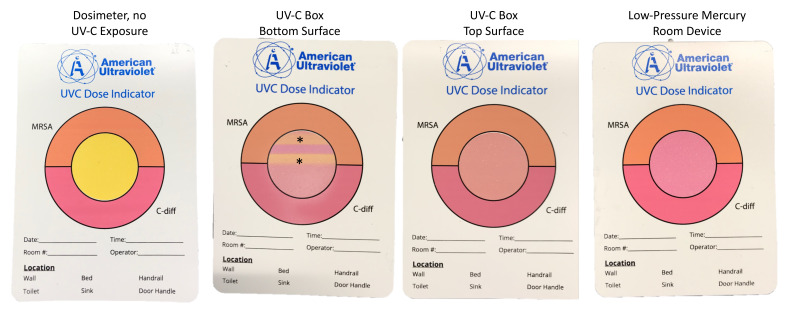
Pictures showing colorimetric indicators placed adjacent to respirators during ultraviolet-C cycles.

**Figure 4** shows the changes in the UV-C colorimetric indicators with each type of UV-C treatment. In the UV-C box, the respirators are placed on narrow slats that are purported to allow UV-C passage. However, as shown in Figure 4, lines present at the site of the slats demonstrate that UV-C penetration was reduced. Both the 1-minute cycle in the UV-C box and the 30-minute cycle with the low-pressure mercury room decontamination device resulted in a color change from yellow (untreated) to pink, indicating delivery of a dose sufficient to reduce *C. difficile* spores.

Figure 5 shows the efficacy of a high-level disinfection cabinet for decontamination of Moldex 1517 respirators inoculated with MRSA and bacteriophage MS2. With 1, 2, and 3 treatment cycles of 21 minutes and with an extended 31-minute cycle, reductions of ≥2.1, ≥3.6, and >6 log_10_ PFU or CFU were achieved for all the test sites. Additional testing with the other 2 respirator types demonstrated similar results for the 3 consecutive 21-minute cycles and for the single extended 31-minute cycle. The 3-cycle treatment was effective in achieving >6-log_10_ PFU or CFU reductions on the Moldex 1517 respirator when the suspension containing 10^6^ PFU of MS2 and 10^6^ CFU of MRSA was sprayed onto the entire inner and outer surface of the Moldex 1517 respirator. No visible changes were observed in any of the respirators after 3 or more cycles of decontamination.

### Efficacy of a multi-purpose high-level disinfection cabinet for decontamination of N95 respirators

**Figure 5.**
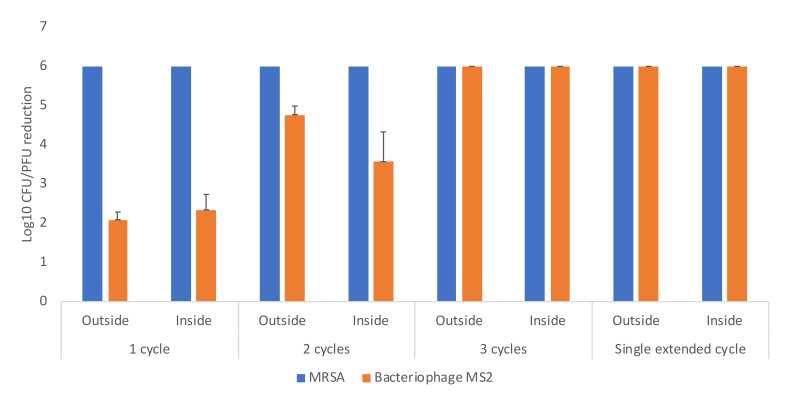
Efficacy of a multi-purpose high-level disinfection cabinet for decontamination or disinfection of methicillin-resistant *Staphylococcus aureus* (MRSA) and bacteriophage MS2 on N95 respirators. 10-μL aliquots containing 10^6^ colony-forming units (CFU) or plaque-forming units (PFU) of the test organisms in the simulated mucous suspension were spread to cover an area of 1-cm^2^ on 3 different areas on the respirator surface (top exterior, edge exterior, and interior) as shown in Figure 1. The respirator was exposed to 1, 2, or 3 22-minute treatment cycles or a single extended cycle of 31 minutes. Error bars indicate standard error.

### Efficacy of dry heat for decontamination of N95 respirators

Dry heat at 70°C for 30 minutes had limited effectiveness against bacteriophages MS2 and Phi6 versus MRSA (<1 log_10_ PFU versus >4 log_10_ CFU respectively, F = 54.7, df = 2, *P*< 0.01).

## DISCUSSION

Shortages of PPE are a grave concern for many healthcare facilities in the setting of the global COVID-19 pandemic. Our findings have important implications for facilities that are considering decontamination of PPE as a potential strategy to maintain adequate supplies. Using a rigorous test method, we found that UV-C reduced contamination of N95 respirators with Phi6 and MS2 bacteriophages and MRSA. However, there was considerable variability in reductions achieved on different respirator brands and on different locations on the respirators. The efficacy on the interior surface of the respirator was reduced in comparison to the outer surface, possibly due to the permeability of the inner surfaces to the liquid suspensions resulting in reduced access by UV-C.

Our results suggest that facilities might consider use of the UV-C box or room decontamination devices to reduce contamination on respirators that will be reused by individuals. However, the levels of reduction did not meet our pre-established criteria for decontamination (ie, ≥3-log_10_ reduction on inoculated respirators), and moreover would not have met a ≥2-log_10_ reduction requirement for decontamination. Thus, the level of reduction would not be adequate to allow shared use of respirators by different individuals.

The high-level disinfection cabinet was more effective than UV-C and provided 2.1 or greater log_10_ reductions in bacteriophage MS2 on both outer and inner surfaces of the respirator with a single cycle. It is notable that this level of reduction is substantially lower than the 6-log_10_ reductions in bacteriophage MS2 achieved on solid carriers in previous studies with this technology [12–13]. Moreover, the single cycle resulted in >6-log_10_ reductions in MRSA and *C. difficile* spores inoculated on the respirator, despite greater UV-C resistance of these organisms on solid surfaces. Taken together, these data suggest that reduction in viral pathogens on N95 respirators might be challenging, in part because the small size of viral particles allows them to penetrate beneath the respirator surface to a greater extent than bacteria resulting in partial protection from technologies such as UV-C and aerosolized peracetic acid. These data also highlight the importance of including viruses in the testing of technologies proposed for N95 respirator decontamination.

With 3 consecutive cycles or an extended cycle, the high-level disinfection cabinet met criteria for disinfection, achieving >6-log_10_ reductions on N95 respirators. The same technology is available as a room decontamination device that would provide additional space for larger quantities of PPE [12]. On solid surfaces, the efficacy of the high-level disinfection cabinet is similar to that reported for hydrogen peroxide vapor, which has also been considered as an option for PPE de-contamination [9, 21]. Given the efficacy of the disinfection cabinet, this type of technology could potentially be used for disinfection of PPE that would be shared among different individuals in the setting of a crisis with inadequate supplies of PPE.

We found that dry heat in an oven at 70°C for 30 minutes had limited effectiveness for decontamination of inoculated respirators. Previous reports suggest that heat can be very effective against viruses in liquid suspension and heated droplets [22–25]. The relative lack of efficacy of heat in our study may be attributable to the use of dry rather than moist heat, inoculation onto respirators rather than hard surfaces or liquid suspensions, and characteristics of the viruses being tested. Lore *et al*. [4] and Heimbuch *et al*. [6] found that moist heat (65°C for 20 minutes) was effective for inactivation of influenza virus. Darnell *et al*. [22] found that heating a liquid suspension with virus particles for 45 minutes at 75°C or 90 minutes at 65°C was effective for inactivation of SARS-CoV. Further studies are needed that include use of moist and dry heat for reduction of viruses inoculated onto respirators.

Our study has several limitations. First, we did not address the concern that decontamination technologies could alter the level of protection provided by PPE [10–11]. Further studies are currently being conducted to evaluate the impact of multiple different decontamination methods on N95 performance such as filtration efficiency. Second, we applied the test organisms in a liquid suspension directly onto the surface of the N95 respirators and spread them over a relatively small surface area with a loop. We cannot exclude the possibility that the technologies would have been more effective if the inoculum was spread out over a larger surface area; spreading of an organism inoculum over a larger surface area has been shown to increase the efficacy of UV-C light [14–15]. It has also been suggested that the method of deposition may influence results, and that methods that more closely mimic droplet and aerosol deposition on respirators should be used [6]. For the disinfection cabinet, we conducted additional experiments in which the inoculum was applied to the entire respirator as a fine spray with similar results (data not shown). Third, the testing was conducted as a laboratory simulation. Additional studies are needed to evaluate decontamination of N95 respirators used in clinical settings. Fourth, UV-C was less effective in our study than has been reported in previous investigations of UV-C for decontamination of influenza virus on respirators [4, 6]. This may be related to differences in methodology or to greater UV-C susceptibility of influenza virus than the bacteriophages studied. Previous studies suggest that viruses vary considerably in susceptibility to UV-C [26]. Fifth, we cannot exclude the possibility that a higher UV-C dose might have resulted in greater efficacy. However, there is evidence that very high UV-C doses may adversely affect N95 respirator performance and structural integrity [10]. Sixth, we only studied 3 brands of N95 respirators and 3 methods of decontamination. We are currently evaluating the effectiveness of several other technologies. Finally, we did not include data on decontamination of other types of PPE. However, testing with surgical face masks yielded similar results (data not shown).

In summary, we found that UV-C reduced contamination of N95 respirators with Phi6 and MS2 bacteriophages and MRSA. However, efficacy varied with different respirator types and with different locations on the respirator. A high-level disinfection cabinet using submicron droplets of aerosolized peracetic acid and hydrogen peroxide was substantially more effective for decontamination of N95 respirators and with 3 consecutive cycles or a single extended cycle achieved >6-log_10_ reductions meeting criteria for disinfection. Further work is urgently needed to determine the impact of decontamination technologies on respirator function.
